# Correction: Effect of bovine leukemia virus (BLV) infection on bovine mammary epithelial cells RNA-seq transcriptome profile

**DOI:** 10.1371/journal.pone.0236912

**Published:** 2020-07-23

**Authors:** The PLOS ONE Staff

The first author’s name is spelled incorrectly. The correct name is: Lucia Martínez Cuesta.

The first author's surname appears incorrectly in the citation. The correct citation is: Martínez Cuesta L, Liron JP, Nieto Farias MV, Dolcini GL, Ceriani MC (2020) Effect of bovine leukemia virus (BLV) infection on bovine mammary epithelial cells RNA-seq transcriptome profile. PLoS ONE 15(6): e0234939. https://doi.org/10.1371/journal.pone.0234939. The publisher apologizes for the error.
