# Supplementary material for: Effect of bovine leukemia virus (BLV) infection on bovine mammary epithelial cells RNA-seq transcriptome profile
Source: PLoS One. 2020 Jun 24;15(6):e0234939. doi: 10.1371/journal.pone.0234939 (PMC7313955; doi:10.1371/journal.pone.0234939)
Supplement: S1 Text — (PDF) [file pone.0234939.s001.pdf]

Data from the sequencing facility before library preparation:

Sample NO.	Sample Name	Novogene ID	Conc (ng/μl)	Vol. (μL)	Amt. (μg)	RIN	Conclusion
1	mact 1	USR18021074	210.49	15.8	3.326	9	Pass
2	mact 2	USR18021075	463.70	18.8	8.718	8	Pass
3	mact3	USR18021906	380.13	18.8	7.146	9	Pass
4	mactblv1	USR18037207	396.58	19.9	7.892	8.9	Pass
5	mactblv2	USR18037204	925.44	11.4	10.550	7.6	Pass
6	mactblv3	USR18021079	679.77	16.2	11.012	9.1	Pass

MACT 1

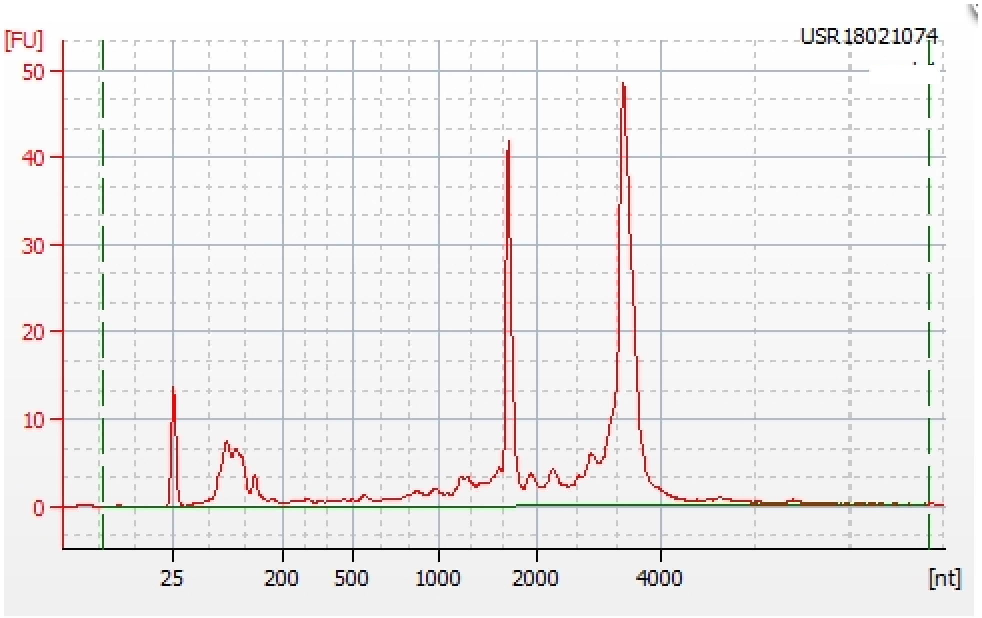

MACT 2

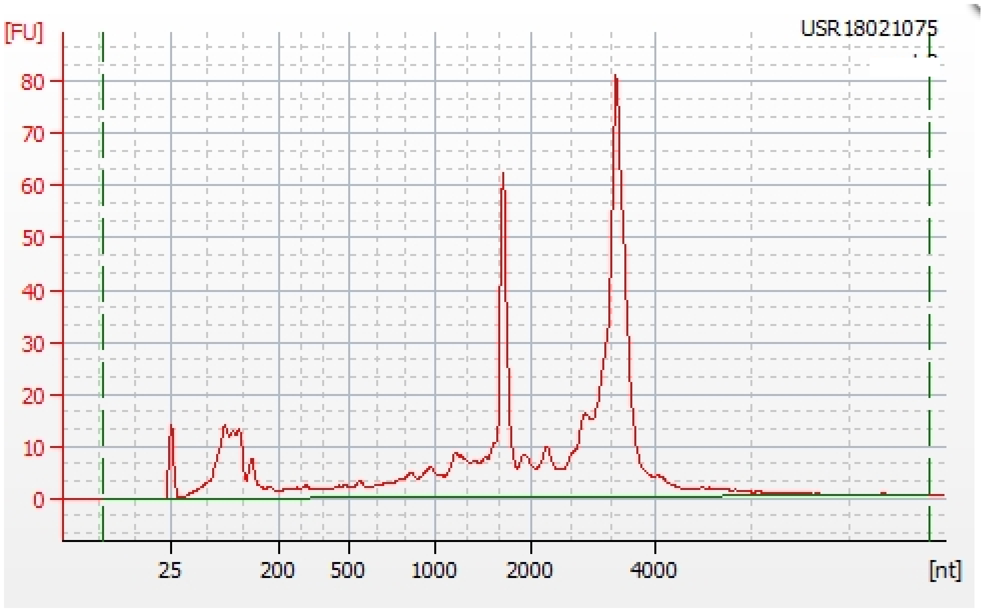

MACT 3

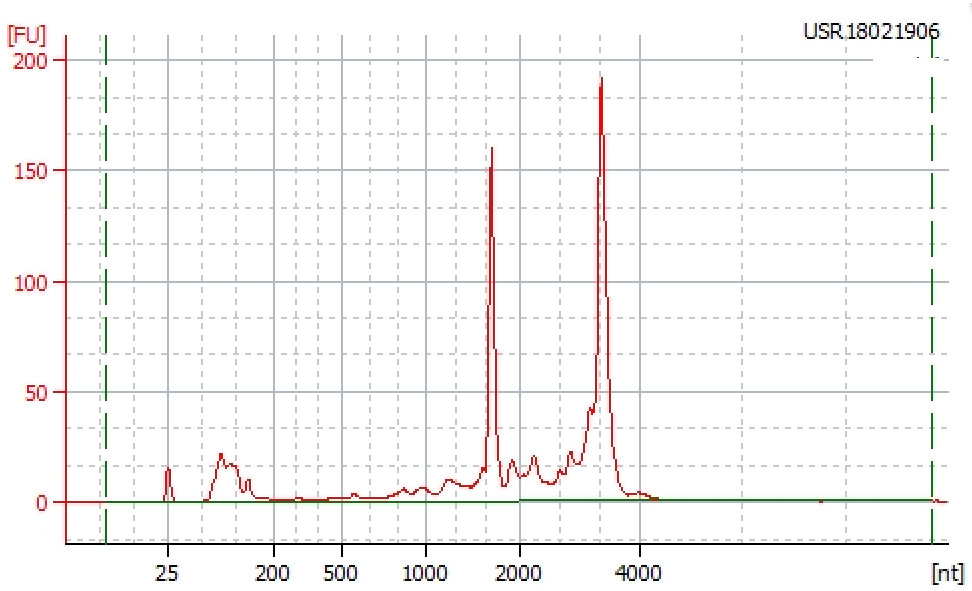

MACT BLV 1

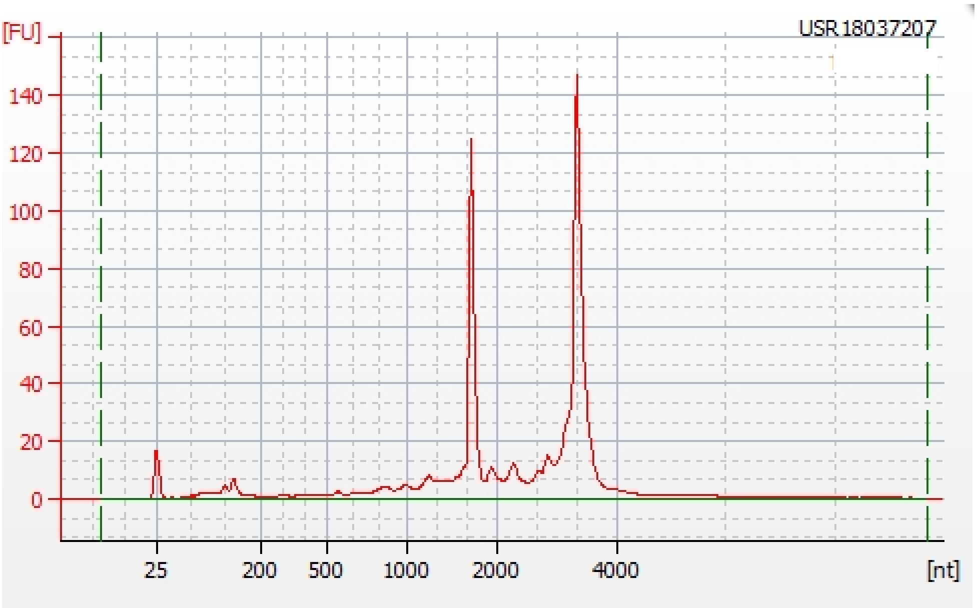

MACTBLV 2

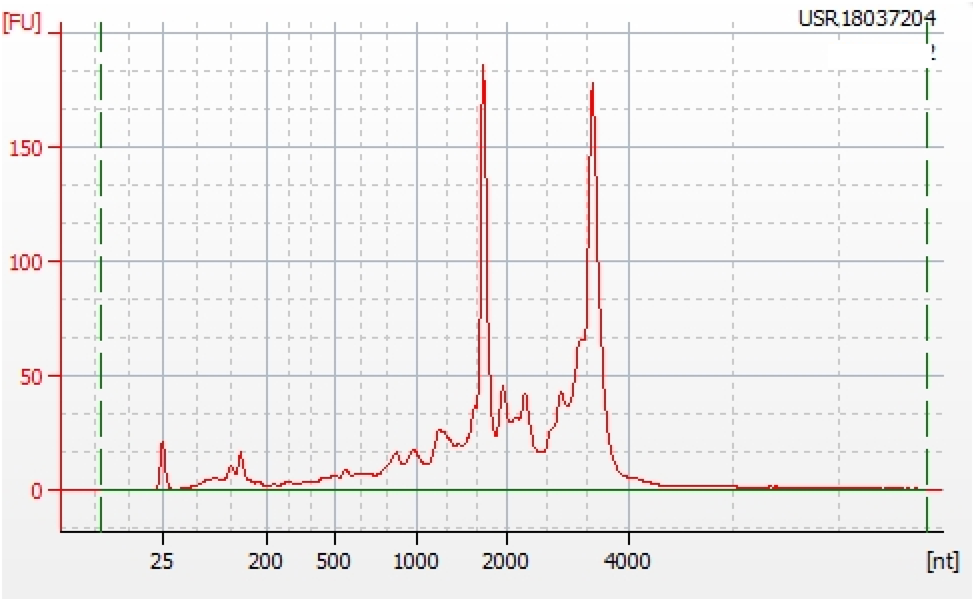

MACTBLV 3

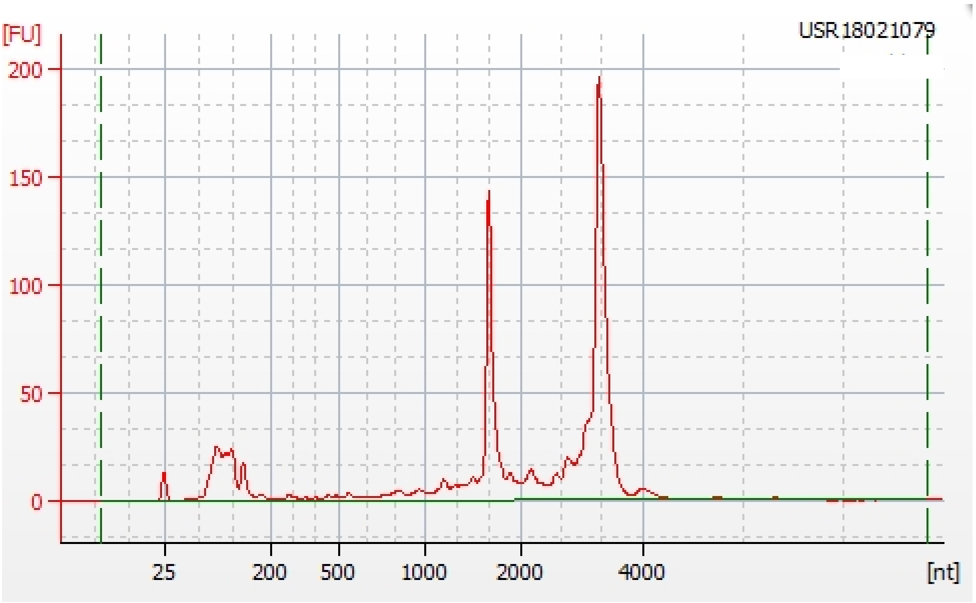
USR18021079
[FU]
200
150
100
50
0
25
200
500
1000
2000
4000
[nt]

RIN OBTAINED IN OUR LAB USING BIOANALYZER BEFORE SENDING THE SAMPLES FOR ANALYSIS

Assay Class:	Eukaryote Total RNA Nano	Created:	1/7/2019 10:48:18 AM
Data Path:	C:\...Eukaryote Total RNA Nano_DE24802750_2019-01-07_10-48-18.xad	Modified:	1/7/2019 11:12:13 AM

Electropherogram Summary

mact 1

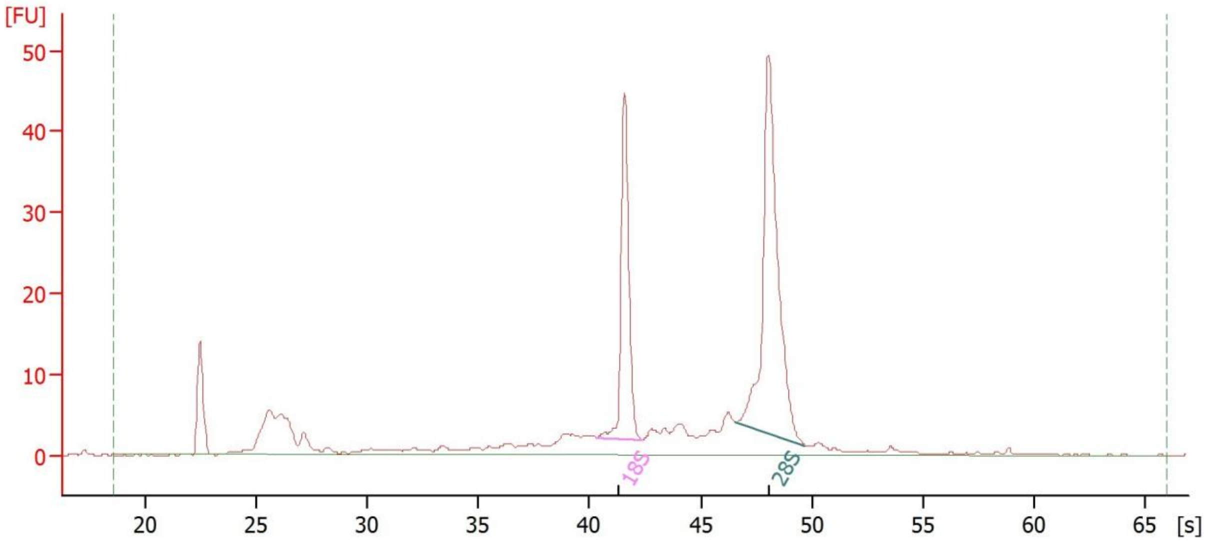

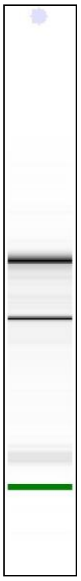

Overall Results for sample 1 : <u>mact 1</u>

RNA Area:	254.0	RNA Integrity Number (RIN):	8.9 (B.02.08)
RNA Concentration:	152 ng/µl	Result Flagging Color:	
rRNA Ratio [28s / 18s]:	1.8	Result Flagging Label:	RIN: 8.90

Fragment table for sample 1 : <u>mact 1</u>

Name	Start Time [s]	End Time [s]	Area	% of total Area
18S	40.32	42.32	40.2	15.8
28S	46.58	49.68	73.3	28.8

 © Copyright 2003 - 2015 Agilent Technologies, Inc.

Assay Class:	Eukaryote Total RNA Nano	Created:	1/7/2019 10:48:18 AM
Data Path:	C:\...Eukaryote Total RNA Nano_DE24802750_2019-01-07_10-48-18.xad	Modified:	1/7/2019 11:12:13 AM

Electropherogram Summary Continued ...

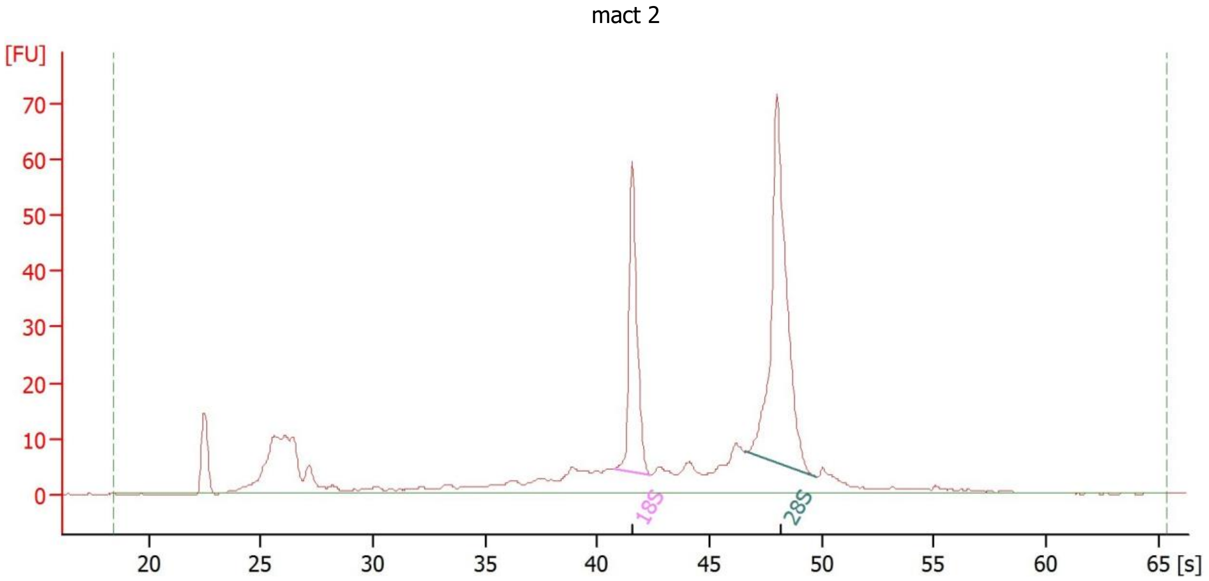

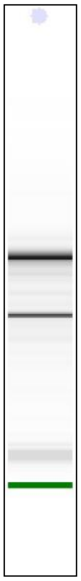

Overall Results for sample 2 : mact 2

RNA Area:	409.8	RNA Integrity Number (RIN):	8.5 (B.02.08)
RNA Concentration:	245 ng/µl	Result Flagging Color:	
rRNA Ratio [28s / 18s]:	1.9	Result Flagging Label:	RIN: 8.50

Fragment table for sample 2 : mact 2

Name	Start Time [s]	End Time [s]	Area	% of total Area
18S	40.74	42.39	56.3	13.7
28S	46.61	49.78	107.7	26.3

 © Copyright 2003 - 2015 Agilent Technologies, Inc.

Assay Class: Eukaryote Total RNA Nano
Data Path: C:\...Eukaryote Total RNA Nano_DE24802750_2019-01-07_10-48-18.xad
Created: 1/7/2019 10:48:18 AM
Modified: 1/7/2019 11:12:13 AM

Electropherogram Summary Continued ...

mact 3

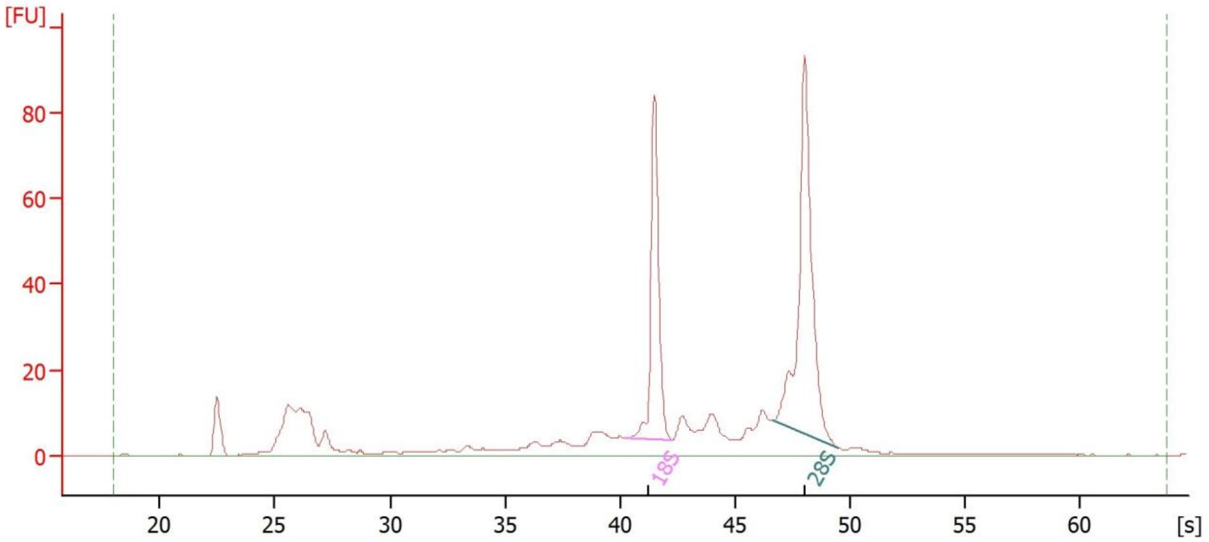

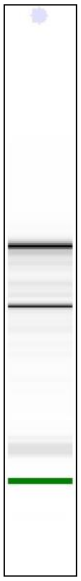

Overall Results for sample 3 : **mact 3**

RNA Area:	455.6	RNA Integrity Number (RIN):	8.7 (B.02.08)
RNA Concentration:	272 ng/µl	Result Flagging Color:	
rRNA Ratio [28s / 18s]:	1.6	Result Flagging Label:	RIN: 8.70

Fragment table for sample3 : **mact 3**

Name	Start Time [s]	End Time [s]	Area	% of total Area
18S	40.20	42.27	73.5	16.1
28S	46.66	49.48	120.0	26.3

 © Copyright 2003 - 2015 Agilent Technologies, Inc.

Assay Class: Eukaryote Total RNA Nano
Data Path: C:\...Eukaryote Total RNA Nano_DE24802750_2019-04-22_10-48-44.xad

Created: 4/22/2019 10:48:43 AM
Modified: 4/22/2019 11:03:18 AM

Electropherogram Summary Continued ...

mactblv 1

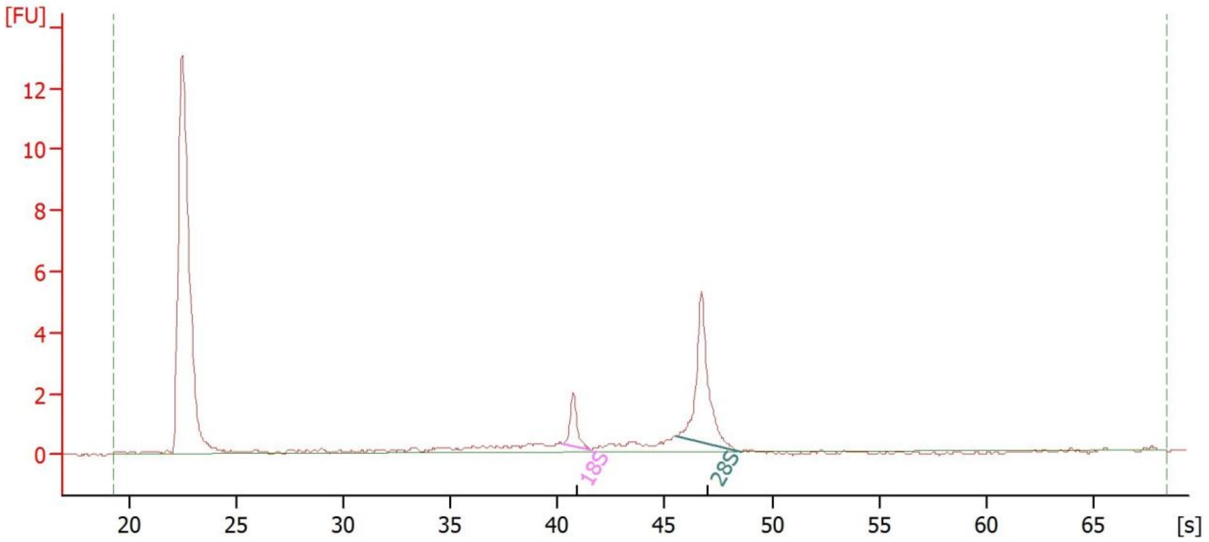

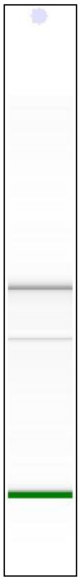

Overall Results for sample 3 : mactblv1

RNA Area:	17.9	RNA Integrity Number (RIN):	8.3 (B.02.08)
RNA Concentration:	488 ng/µl	Result Flagging Color:	
rRNA Ratio [28s / 18s]:	4.2	Result Flagging Label:	RIN: 8.30

Fragment table for sample 3 : mactblv1

Name	Start Time [s]	End Time [s]	Area	% of total Area
18S	40.24	41.53	1.6	9.1
28S	45.50	48.52	6.9	38.6

 © Copyright 2003 - 2015 Agilent Technologies, Inc.

Assay Class:	Eukaryote Total RNA Nano	Created:	4/22/2019 10:48:43 AM
Data Path:	C:\...Eukaryote Total RNA Nano_DE24802750_2019-04-22_10-48-44.xad	Modified:	4/22/2019 11:03:18 AM

Electropherogram Summary Continued ...

mactblv 2

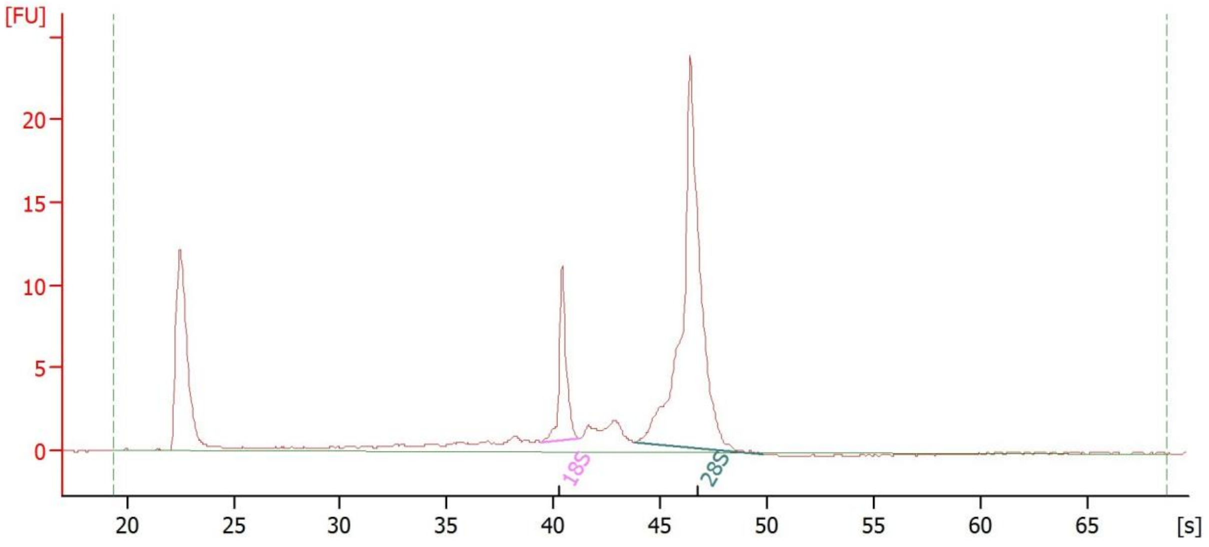

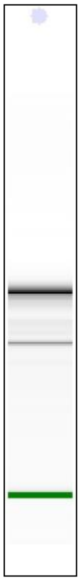

Overall Results for sample 2 : <u>mactblv2</u>

RNA Area:	91.8	RNA Integrity Number (RIN):	9.1 (B.02.08)
RNA Concentration:	2,511 ng/µl	Result Flagging Color:	
rRNA Ratio [28s / 18s]:	5.0	Result Flagging Label:	RIN: 9.10

Fragment table for sample 2 : <u>mactblv2</u>

Name	Start Time [s]	End Time [s]	Area	% of total Area
18S	39.38	41.17	10.0	10.9
28S	43.81	49.78	50.3	54.8

© Copyright 2003 - 2015 Agilent Technologies, Inc.

Assay Class: Eukaryote Total RNA Nano
Data Path: C:\...Eukaryote Total RNA Nano_DE24802750_2019-01-07_10-48-18.xad

Created: 1/7/2019 10:48:18 AM
Modified: 1/7/2019 11:12:13 AM

Electropherogram Summary Continued ...

mactblv 3

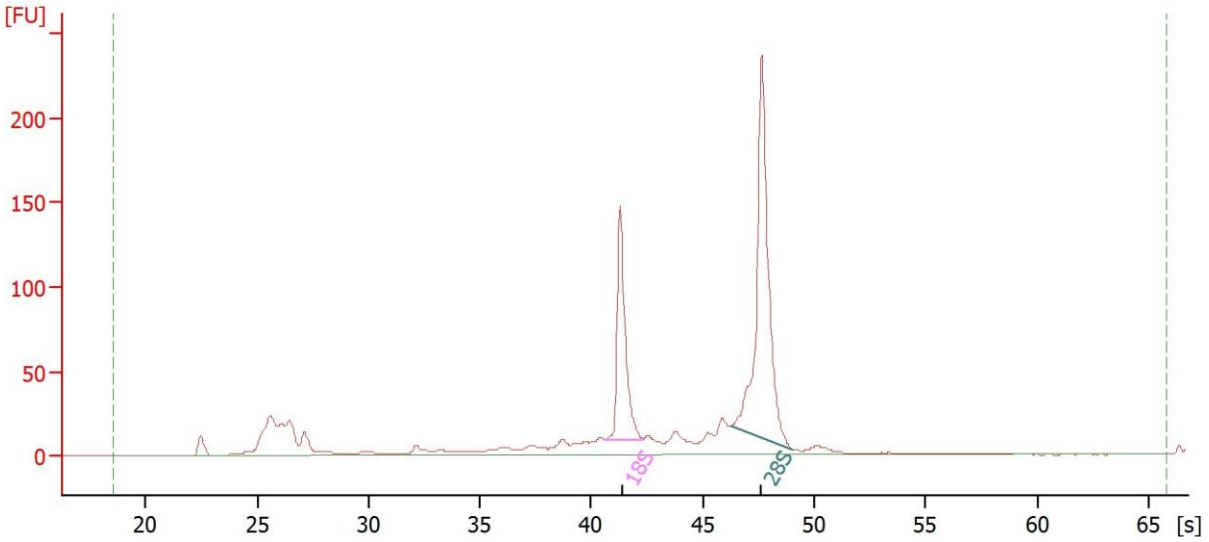

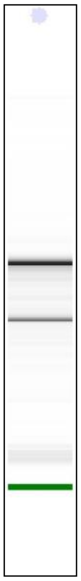

Overall Results for sample 11 : **mactblv 3**

RNA Area:	925.2	RNA Integrity Number (RIN):	9.1 (B.02.08)
RNA Concentration:	552 ng/µl	Result Flagging Color:	
rRNA Ratio [28s / 18s]:	2.1	Result Flagging Label:	RIN: 9.10

Fragment table for sample 11 : **mactblv 3**

Name	Start Time [s]	End Time [s]	Area	% of total Area
18S	40.61	42.28	137.8	14.9
28S	46.24	49.05	293.3	31.7

 © Copyright 2003 - 2015 Agilent Technologies, Inc.
