# Supplementary material for: Effect of bovine leukemia virus (BLV) infection on bovine mammary epithelial cells RNA-seq transcriptome profile
Source: PLoS One. 2020 Jun 24;15(6):e0234939. doi: 10.1371/journal.pone.0234939 (PMC7313955; doi:10.1371/journal.pone.0234939)
Supplement: S1 Table — (DOCX) [file pone.0234939.s002.docx]

**S1 Table. Most differentially expressed genes between MAC-T and MAC-T BLV.**

| **Ensembl ID** | **log2 FC** | **Adjusted p-value** | **Name** | **Description** |
| --- | --- | --- | --- | --- |
| ENSBTAG00000037527 | 7.5 | 4.42E-08 | OAS1Z | 2',5'-oligoadenylate synthetase 1 |
| ENSBTAG00000005774 | 7 | 1.11E-04 | VRTN | vertebrae development associated |
| ENSBTAG00000044063 | 6.73 | 3.21E-05 | B4GALT6 | beta-1,4-galactosyltransferase 6 |
| ENSBTAG00000021282 | 6.46 | 6.54E-04 | SGCE | sarcoglycan epsilon |
| ENSBTAG00000000720 | 6.35 | 1.21E-03 | CTSL | cathepsin L |
| ENSBTAG00000008471 | 6.29 | 1.18E-08 | MX2 | MX dynamin like GTPase 2 |
| ENSBTAG00000013848 | 5.93 | 1.62E-02 | ADGRD1 | adhesion G protein-coupled receptor D1 |
| ENSBTAG00000044161 | 5.78 | 1.01E-02 | NMU | neuromedin U |
| ENSBTAG00000053807 | 5.77 | 1.01E-13 | OAS1X | 2',5'-oligoadenylate synthetase 1 |
| ENSBTAG00000003403 | 5.75 | 2.16E-03 | PADI2 | peptidyl arginine deiminase 2 |
| ENSBTAG00000014707 | 5.75 | 2.67E-39 | ISG15 | ISG15 ubiquitin-like modifier |
| 519280 | 5.69 | 1.42E-02 | None | None |
| ENSBTAG00000019772 | 5.67 | 1.45E-02 | OXTR | oxytocin receptor |
| 615581 | 5.43 | 7.82E-04 | None | None |
| ENSBTAG00000016061 | 5.31 | 1.47E-10 | RSAD2 | radical S-adenosyl methionine domain containing 2 |
| ENSBTAG00000039861 | 5.26 | 5.55E-22 | OAS1Y | 2',5'-oligoadenylate synthetase 1, 40/46kDa |
| ENSBTAG00000014628 | 5.23 | 5.55E-22 | OAS2 | 2'-5'-oligoadenylate synthetase 2 |
| ENSBTAG00000016169 | 5.18 | 6.07E-04 | ID1 | inhibitor of DNA binding 1, HLH protein |
| ENSBTAG00000018966 | 5.16 | 2.82E-03 | PLCE1 | phospholipase C epsilon 1 |
| ENSBTAG00000045940 | 5.15 | 1.71E-02 | MGC148328 | Bos taurus uncharacterized LOC783058 (MGC148328) mRNA |
| ENSBTAG00000007554 | 4.98 | 1.62E-18 | IFI6 | interferon alpha inducible protein 6 |
| ENSBTAG00000030913 | 4.81 | 3.14E-21 | MX1 | MX dynamin like GTPase 1 |
| ENSBTAG00000019630 | 4.67 | 1.15E-05 | RGL1 | ral guanine nucleotide dissociation stimulator like 1 |
| ENSBTAG00000016355 | 4.25 | 1.53E-04 | UVRAG | UV radiation resistance associated |
| ENSBTAG00000038103 | 4.24 | 1.96E-02 | DPPA4 | developmental pluripotency associated 4 |
| ENSBTAG00000011343 | 3.9 | 1.64E-05 | XAF1 | XIAP associated factor 1 |
| ENSBTAG00000006846 | 3.88 | 2.83E-10 | LGALS9 | galectin 9 |
| ENSBTAG00000016486 | 3.82 | 2.23E-02 | SORBS2 | sorbin and SH3 domain containing 2 |
| ENSBTAG00000017024 | 3.77 | 2.84E-06 | PPARGC1A | PPARG coactivator 1 alpha |
| ENSBTAG00000020195 | 3.71 | 2.67E-06 | ANKRD34C | ankyrin repeat domain 34C |
| ENSBTAG00000003152 | 3.69 | 6.74E-07 | IFI27 | putative ISG12(a) protein |
| ENSBTAG00000053649 | 3.67 | 1.05E-02 | CCL5 | C-C motif chemokine ligand 5 |
| ENSBTAG00000002348 | 3.64 | 4.39E-02 | SLC4A4 | solute carrier family 4 member 4 |
| ENSBTAG00000019716 | 3.61 | 4.98E-04 | CXCL8 | C-X-C motif chemokine ligand 8 |
| ENSBTAG00000002998 | 3.57 | 2.00E-04 | MELTF | melanotransferrin |
| ENSBTAG00000001969 | 3.56 | 3.04E-03 | CCDC39 | coiled-coil domain containing 39 |
| ENSBTAG00000047380 | 3.51 | 8.53E-03 | CALCB | calcitonin related polypeptide beta |
| ENSBTAG00000052025 | 3.46 | 1.29E-02 |  |  |
| ENSBTAG00000012656 | 3.35 | 1.91E-04 | OVOL1 | ovo like transcriptional repressor 1 |
| ENSBTAG00000021588 | 3.3 | 1.53E-04 | SCG2 | secretogranin II |
| ENSBTAG00000019293 | -3.23 | 5.50E-05 | EGR3 | early growth response 3 |
| ENSBTAG00000020676 | -3.36 | 1.03E-08 | MMP9 | matrix metallopeptidase 9 |
| ENSBTAG00000016121 | -3.49 | 6.11E-07 | KRT75 | keratin 75 |
| ENSBTAG00000014835 | -3.85 | 1.70E-03 | SPARC | secreted protein acidic and cysteine rich |
| ENSBTAG00000015059 | -3.85 | 2.66E-03 | MMP13 | matrix metallopeptidase 13 |
| ENSBTAG00000007196 | -3.96 | 1.94E-02 | TAGLN | transgelin |
| ENSBTAG00000019070 | -4.18 | 6.33E-03 | PMP22 | peripheral myelin protein 22 |
| ENSBTAG00000038662 | -5.63 | 8.53E-03 | GJB6 | gap junction protein beta 6 |
| ENSBTAG00000020985 | -6.34 | 1.06E-02 | TYRP1 | tyrosinase related protein 1 |
| ENSBTAG00000014665 | -8.47 | 1.08E-05 | ADAMTS2 | ADAM metallopeptidase with thrombospondin type 1 motif 2 |
