# Supplementary material for: Effect of bovine leukemia virus (BLV) infection on bovine mammary epithelial cells RNA-seq transcriptome profile
Source: PLoS One. 2020 Jun 24;15(6):e0234939. doi: 10.1371/journal.pone.0234939 (PMC7313955; doi:10.1371/journal.pone.0234939)
Supplement: S2 Table — (DOCX) [file pone.0234939.s003.docx]

**S2 Table. Summary of the sequences aligned with BLV reference genome AP018032.**

| **Sample** | **Unique alignment** | **Multiple alignment** | **No alignment** |
| --- | --- | --- | --- |
| **MAC-T 1** | 0 | 1 | 0 |
| **MAC-T 2** | 5 | 0 | 0 |
| **MAC-T 3** | 0 | 0 | 0 |
| **MAC-T BLV 1** | 788446 | 49255 | 28663790 |
| **MAC-T BLV 2** | 679264 | 41613 | 28544425 |
| **MAC-T BLV 3** | 557234 | 40672 | 26584493 |
